# Evolution of Blood Safety in Switzerland over the Last 25 Years for HIV, HCV, HBV and *Treponema pallidum*

**DOI:** 10.3390/v14122611

**Published:** 2022-11-23

**Authors:** Christoph Niederhauser, Caroline Tinguely, Martin Stolz, Michael Vock, Soraya Amar El Dusouqui, Peter Gowland

**Affiliations:** 1 Interregional Blood Transfusion SRC, 3008 Bern, Switzerland; 2 Institute for Infectious Disease, University of Bern, 3001 Bern, Switzerland; 3 Faculté de Biologie et de Médecine, Université de Lausanne, 1015 Lausanne, Switzerland; 4 Institute of Mathematical Statistics and Actuarial Science, University of Bern, 3012 Bern, Switzerland; 5 Swiss Transfusion SRC, 3008 Bern, Switzerland

**Keywords:** blood donation, evolution of the screening strategies, nucleic acid amplification technology (NAT), safety of labile blood components

## Abstract

During the last few decades, efforts to increase the safety of blood and blood products have mainly focused on preventing the viral infections HCV, HIV, HBV and *Treponema pallidum*. The evolution of these approaches and the achieved increase in safety is shown for the last 25 years in Switzerland. In detail, the prevalences and incidences of the infection disease and the theoretical estimated residual risks (RR) of these blood-borne infections are presented. Prevalences, incidences and, in particular, the RR have decreased considerably over the last 25 years. This was achieved primarily by the adoption of strict criteria for the selection of blood donors, refined questionnaires, the introduction of increasingly sensitive serological screening tests and the implementation of nucleic acid testing (NAT) for these blood-borne pathogens. These NAT assays have significantly shortened the window period between infection and the first detection of the infectious agent in the blood of an infected individual. A form of “real life” comparison or confirmation is provided by the reported lookback procedures (LBP) and the haemovigilance data of the Swiss competent authority, Swissmedic. These data are in agreement, and thus support the very low prevalences, incidences and RR.

## 1. Introduction

The Blood Transfusion Service from the Swiss Red Cross (BTS SRC) is an institution affiliated to the Swiss Red Cross (SRC). In 1951, the Swiss national government commissioned the SRC to build up a blood transfusion service to supply the health community of Switzerland with sufficient blood and blood components. This supply relies solely on donations from voluntary, unpaid blood donors.

There are currently 11 regional blood transfusion services (BTS) responsible for organizing the blood collection and testing the blood products. The national umbrella organization BTS SRC is responsible for the national guidelines and recommendations, including the screening algorithms, test confirmation and lookback procedures (LBP) [1].

A national reference laboratory for the infectious disease markers (NRL) for BTS SRC was also set up in 1996 to perform mandatory confirmation tests on all repeat reactive blood donations. The Interregional Blood Transfusion SRC (IRB) is currently mandated to run the NRL for BTS SRC. The BTS SRC, NRL, together with internal working groups, are responsible for formulating the national guidelines for blood donation testing algorithms, LBP, post-donation information (PDI) and blood donor deferral policies in Switzerland [1].

Over the last 25 years in Switzerland, similar to all other industrialised countries, the safety of labile blood components for transfusion has increased significantly. This was achieved, on the one hand, by adopting strict donor selection criteria and refined donor questionnaires and, on the other hand, by introducing specific and increasingly sensitive serological screening tests for the known transfusion transmissible infections (TTI) and by the successive introduction of nucleic acid testing (NAT) for these major blood-borne pathogens. These assays have significantly shortened the window period (WP) between infection and the first detection of the infectious agent in the blood of an infected individual. As a consequence of these measures, the risk of the introduction of the blood-borne viruses human immunodeficiency virus (HIV), hepatitis C virus (HCV) or hepatitis B virus (HBV) and *Treponema pallidum,* the causative agent of syphilis, entering the blood supply has incrementally declined. The current risks of such TTI are far too low to directly assess the risk via prospective follow-up and LBP of potentially infected recipients. Therefore, mathematical models have been developed to estimate the theoretical residual risks (RR). Despite these measures, the risk can never be zero [2,3,4,5]. These models are based on the incidence rates of HIV, HCV, HBV and syphilis and on the length of the WP of these corresponding infections, i.e., the temporal gap, spanning from the time point of infection to the first detectable specific infection markers [2,3,4,5]. The introduction of NAT for HCV, HIV and HBV has considerably shortened the WP for these major viruses; thus, the corresponding RR has markedly decreased [6,7,8,9].

Blood collected in the viraemic phase of the WP can potentially be transmitted to the transfused recipient if the viral load is below the detection limit of the used screening assay. Hence, the sensitivity of the screening assays is crucial in determining the length of the WP and, consequently, in estimating the theoretical residual risk. 

## 2. Materials and Methods

### 2.1. Blood Donors

Blood donations from 100% voluntary, unpaid blood donors collected at regional BTS between 1996 and 2021 were included. The donors were aged between 18 and 65 years or up to 75 years with regular medical examinations. All donations were tested according to the valid specifications at the time, documented in the guidelines of the BTS SRC [1].

### 2.2. Evolution of the Screening Strategy in Switzerland

In the beginning, only serological tests were used; however, NAT technology was introduced as a mandatory test for HCV in 1999, for HIV in 2002 and for HBV in 2010. Syphilis is only tested serologically. Prior to 1999, Swiss blood donors were tested only with serological assays for anti-HIV antibodies, anti-HCV antibodies and HBV surface antigen (HBsAg) and antibodies against *Treponema pallidum* and increased alanine aminotransferase (ALAT) activity.

The evolution of the NAT algorithm and platforms used is presented in Table 1 for IRB as an example from Switzerland. Most testing centres initially used pools of up to 49 donations for HCV-NAT. In 2001, an HIV TTI event was confirmed in Switzerland, and, as a consequence of this event, HIV-NAT was declared mandatory too. The sensitivity limit at that time for HIV-1 was 10,000 IU/mL in an individual donation. After intensive discussion with our governing health authority, Swissmedic, it was decided, in 2010, to abolish ALAT testing as a surrogate marker of HBV infection and to replace it with HBV-NAT. At this time, it was also decided not to implement anti-HBc as a screening marker for blood donations. IRB is the major testing laboratory and currently analyses around 50% of all Swiss blood donations. IRB began individual donation NAT (ID-NAT) in 2007 for HCV, HIV and HBV during the introduction of the multiplex Grifols Tigris system. When dual targeting for HIV-1 was recommended in 2015, all Swiss test centres switched to multiplex ID-NAT. Two highly automated test systems are currently used: the Roche Diagnostics cobas MPX test on the 6800/8800 analyser and Grifols Procleix assay on the Panther analyser. The evolution of the viral detection sensitivities that are achieved with the implementation of new ID-NAT assays have played an important role in estimating the theoretical RR of these blood-borne viruses.

### 2.3. The Current Mandatory Testing Strategy in Switzerland

For HCV, anti-HCV antibody assay and HCV ID-NAT; for HIV, anti-HIV 1/2 p24 antigen/antibody combination assay and HIV ID-NAT; for HBV, HBsAg assay and HBV ID-NAT; and for syphilis, a *T. pallidum* particle or hemagglutination test (TTPA) or *T. pallidum* enzyme-linked immunoassay (EIA) [1]. In addition to the guidelines of BTS SRC, Swissmedic has defined the minimal NAT detection limits for all these blood-borne viruses. They are published in an Appendix of the Ordinance of 14 November 2018 on Licensing in the medicinal products sector [10]. The method selected and the design of the screening procedure require the reliable detection of 500 IU/mL HIV-1 RNA and 50 IU/mL HCV RNA in a single blood donation (based on the corresponding WHO standards). The detection limit for HBV was set at 25 IU/mL HBV DNA. These detection limits are easily achieved with the current NAT systems.

### 2.4. Confirmation of Screening Reactive Samples

Confirmation of initial reactive samples is performed, according to the BTS guidelines [1] For both serological and/or NAT reactive blood donor samples, specific algorithms for each infectious disease agent exist.

### 2.5. Theoretical Residual Risk Estimations

Repeat donors (RD) are donors who had previously donated blood at a given regional BTS. Index cases are confirmed positive blood donors whose previous donation had been negative for a specific disease marker. Incidence data were calculated using the following formula: incident cases/number of repeat donations x mean number of donations per year and donor. Due to the lack of data on the specific inter-donation interval, we assessed the average number of donations per year and donor. For HBV, the incidence data were adjusted by a factor of 2.38, according to the model of Korelitz [4]. In 2009, the Korelitz factor was adjusted downwards to 1.5, as was suggested in the publication of Weusten and colleagues [11].

The estimated RR was determined using the following formula: incidence x window period in days/365 or 366, respectively [5]. The serological window periods used for HIV, HCV and HBV were 22, 66 and 59 days, respectively, and the NAT-WP for HCV and HIV were 21 and 11 days at the time of the implementation of MP-NAT, respectively [12,13]. With the implementation of the HCV-NAT in 1999 and HIV-NAT in 2002 and partial implementation of HBV NAT in 2007, the NAT-WP levels were further reduced. Since the mandatory implementation of the highly sensitive ID-NAT in 2016, the HCV, HIV and HBV WP were further reduced to 4, 6 and 15 days, respectively. These are only average values as they take into account the different testing systems used at the various test centres. Galel and co-authors recently specified a rather conservative WP calculation since the introduction of mandatory ID-NAT: 4 for HCV, 6 for HIV and 15 for HBV [14].

### 2.6. Lookback Procedures (LBP)

LBP for either donor- or patient-related cases have been in place since 1996 in Switzerland. They are an important tool to prove or rule out a possible TTI event in blood product recipients. The exact procedures required by Swissmedic and the Federal Office of Public Health (FOPH) for these LBP are described in BTS SRC guidelines and regulations [1].

## 3. Results

### 3.1. Blood Donors

**Figure 1:**
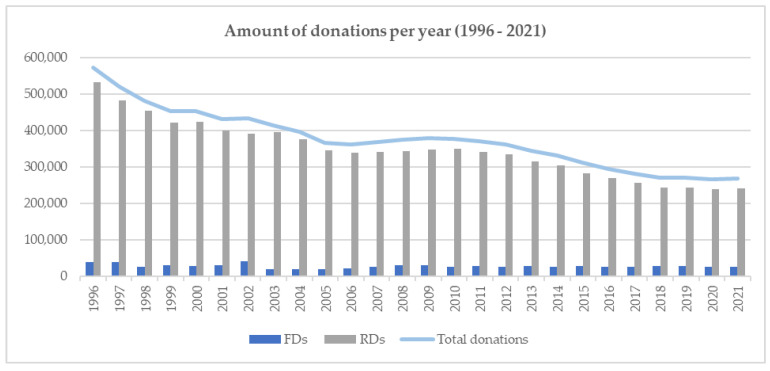
Amount of total donations and donations from RD and FD from 1996–2021.

Between 1996 and 2021, 9,753,402 blood donations from 100% non-renumerated blood donors were collected in Switzerland. Of these, 92.5% (9,020,349 donations) were collected from repeat donors (RD) and 7.5% (733,053 donations) from first-time donors (FD) (Figure 1). There has been a constant decrease in the number of donations collected over the current time period, especially apparent for RD.

**Figure 2:**
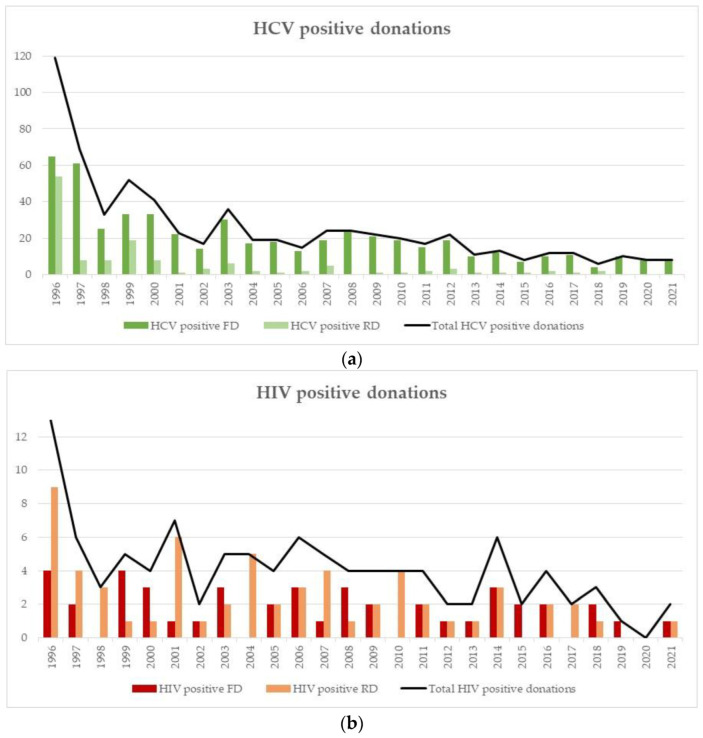

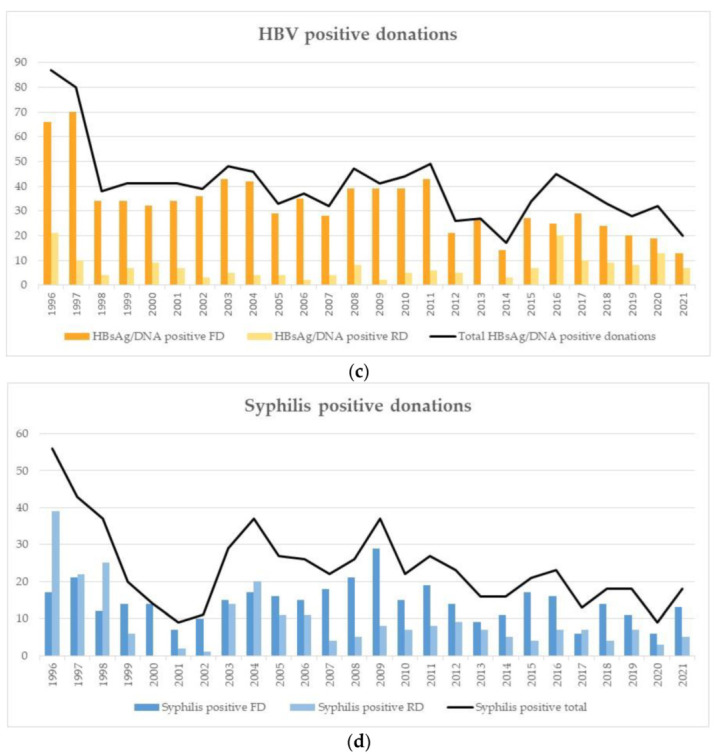
(**a**) HCV confirmed positive donations for RDs and FDs. (**b**) HIV confirmed positive donations for RDs and FDs. (**c**) HBV confirmed positive donations for RDs and FDs. (**d**) Syphilis confirmed positive donations for RDs and FDs.

The confirmed positive cases for the three major viruses, HCV, HIV and HBV, and syphilis over the last 25 years are presented in Figure 2a–d. For all the three viruses between 1996 and 2000, a substantial decrease in cases was observed (Figure 2a–c). In total, over the whole 25 years the following positive FD and RD for HCV, HIV, HBV and syphilis were detected: 528 FD and 132 RD for HCV; 44 FD and 61 RD for HIV; 862 FD and 183 RD for HBV; and 377 FD and 241 RD for syphilis.

The number of HCV confirmed positive cases was highest in 1996 with 119 cases, and since then the number has decreased constantly. From 2013 to 2021, only 6 to 13 confirmed HCV positive cases per year were detected (Figure 2a). The number of HIV confirmed positive donations was also highest in 1996 with 13 cases. Since 1997, the number of cases is stable between zero and seven cases per year (Figure 2b). For HBV, the number of confirmed positive cases was highest in 1996 and 1997 with 87 and 80, respectively. Between 1999 and 2011, relatively high numbers of HBV cases were still being reported (32–48 cases/year), whereas from 2012 to 2014 a decrease was seen, (17–27 cases /year). In 2015 and especially in 2016, coinciding with the introduction of the highly sensitive HBV ID-NAT, a clear increase was observed (45 HBV cases in 2016) (Figure 2c). Since 2017, the number of HBV cases decreased again. The number of confirmed syphilis cases has been far less stable than those observed with the blood-borne viruses. The highest number of confirmed positive syphilis cases was observed in 1996 and 1997 (56 and 43, respectively). Between 1998 and 2002, the number of positive cases was quite low (between 9–20 cases) and thereafter again a slight increase until 2009 with a peak of 38 cases. Thereafter, the number decreased again and currently fluctuates between 9 to 27 cases (Figure 2d).

### 3.2. Incidences in RD

**Figure 3:**
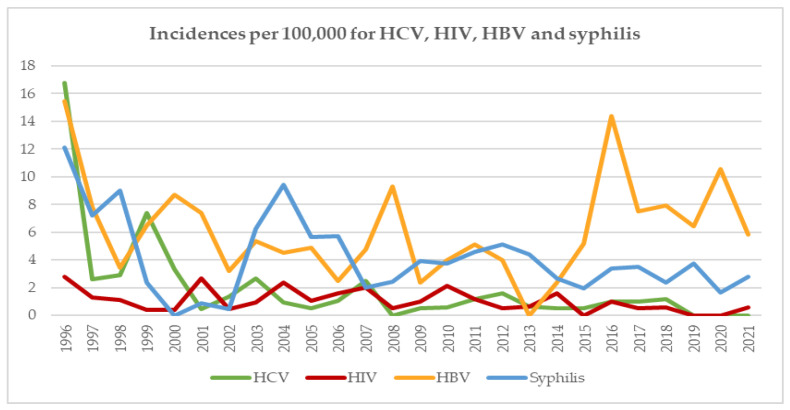
Incidences per 100,000 RD of HCV, HIV, HBV and syphilis from 1996–2021.

The incidences in RD for HCV, HIV and HBV decreased between 1996 and 1999 and have since remained relatively stable (Figure 3). In the last three years of this analysis (2019–2021), no HCV and only one HIV confirmed case was detected. An increase in the incidence for HBV with a peak during 2016 was due to the implementation of the highly sensitive HBV ID-NAT. This change of the testing technology allowed the detection of numerous occult hepatitis B virus (OBI) donors, which had previously been missed with the less sensitive pool NAT technology. Since 2016, there has been a slow decline in the number of OBI cases, and, therefore, there has also been a corresponding decrease in HBV incidence.

### 3.3. Prevalences in FD

**Figure 4:**
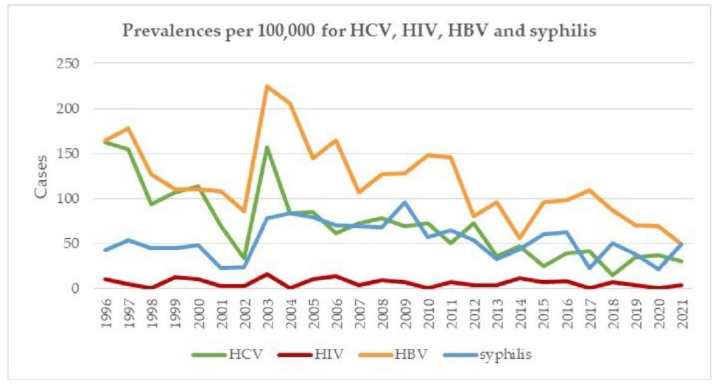
HCV, HBV, HIV and syphilis prevalences per 100,000 FD from 1996–2021.

HCV, HIV, HBV and syphilis prevalences per 100,000 donations from FD between 1996 and 2021 are presented in Figure 4. For HCV, a downward trend from approximately 120 cases per 100,000 donations down to 30 cases was observed. The prevalences for HIV and syphilis show no clear trend over these 25 years, with a relatively large variation. For HIV, in addition, there were no cases in 1998, 2004, 2010, 2017 and 2020, whereas for HBV, a slight downward trend from approximately 130 to 80 cases per 100,000 was observed.

### 3.4. Residual Risks (RR)

**Figure 5:**
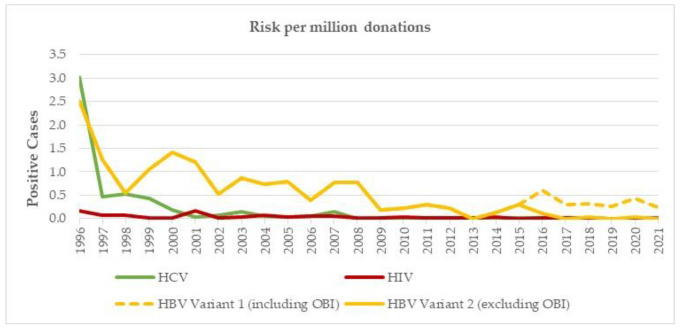
Theoretical estimated RR for HCV, HIV and HBV from 1996 to 2021. HBV has two models after 2015, one with the OBI cases and one without OBI cases.

The theoretical estimated RR for the three major viruses are presented in Table 2 and Figure 5. The 95% confidence intervals for the inverse of the RR are presented in Table 1 of Appendix A; these are based on the Clopper–Pearson confidence interval for the probability of an individual repeat donation being positive. The RR for HCV drastically reduced from 1:(3.3 × 10^4^) donations in 1996 to 1:(1.6 × 10^7^) donations in 2018. Since then, no single HCV positive donation has been observed, and, therefore, the estimated RR for 2019, 2020 and 2021 is exactly 0 (with an upper confidence limit of about 1:(4.6 × 10^6^), according to Appendix A, Table A1).

For HIV, the RR decreased from 1:(5.9 × 10^5^) donations in 1996 to 1:(1.1 × 10^7^) donations in 2021. For HBV, only a moderate decrease from 1:(7.4 × 10^4^) donations in 1996 to 1:(3.2 × 10^5^) donations in 2021 was observed. This decrease can even be observed in the calculated model that also included the OBI cases (i.e., HBsAg negative but positive for HBV DNA). It is assumed that HBV cases with very low viral loads (≤5 IU/mL) are rarely infectious. If the model is modified to remove these OBI cases with low viral loads, then the RR estimations in 2020 is 1 case in 3 × 10^6^ donations. The calculation is in good agreement with the actual observed TTI for HBV, as no cases have been observed since the introduction of the HBV-NAT in 2010.

### 3.5. Lookback Procedures (LB)

**Figure 6:**
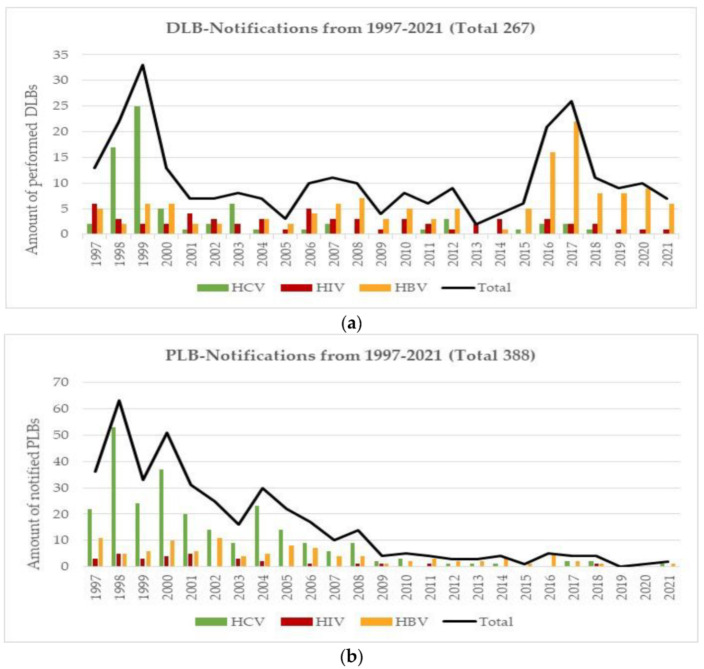
(**a**) DLB notifications between 1997–2021. (**b**) PLB notifications between 1997–2021.

Very few TTI cases have been registered in Switzerland. Most, if not all, occurred before the implementation of the corresponding NAT technology. LB have been performed, recorded and evaluated in Switzerland since 1999. There are two types of LB procedures: donor-related LB (DLB) and patient-related LB (PLB). Similar to the general decrease in confirmed positive donor cases, the corresponding decrease in incidence and prevalence and the RR calculated risks decrease, there has also been an clear reduction in the number of DLB and PLB cases (Figure 6a,b). Here, again, with the implementation of the highly sensitive HBV NAT and the detection of the HBV OBI cases, the DLB increased for 2 years (2016–2017) but has since decreased during 2018 to 2021. The number of PLB has also dramatically declined over this time period. This decrease suggests that if a hospital transmission is suspected, then the involved blood products are highly unlikely to be the source of the infection. For syphilis, no LB were carried out because, on the one hand, only antibodies of a past syphilis infection were measured, and, on the other hand, treponemal spirochetes have a short survival time in blood.

Lookback outcomes: A single HCV case was suspected in 2000 but it could not be confirmed whether it was caused by any contaminated blood product. Between the introduction of anti-HCV antibody testing and before the introduction of HCV-NAT in 1999, there were several HCV TTI cases suspected to be caused by contaminated blood but none were totally confirmed. During the early days for anti-HCV screening prior to 1992, there were >30 documented cases. The last confirmed HIV-1 TTI case was reported in 2001. In 2002, HIV-NAT was declared compulsory, and since then no further HIV TTI case has been reported. Before the introduction of HBV NAT, several TTI cases were suspected but not fully confirmed. One probable HBV TTI case was reported in 2005 and three further cases were documented in 2008 and 2009, just before the mandatory implementation of the HBV-NAT. Two of these cases stemmed from one split RBC [15,16]. After the implementation of the highly sensitive HBV ID-NAT in 2016, eight DLB cases were initiated, and they were all from donors with confirmed but extremely low HBV viral loads. Unfortunately, data and sample material from the implicated recipients could not be obtained despite an extensive search through patient records and stored samples.

### 3.6. NAT Yields 

In low-prevalence countries, such as Switzerland, NAT yield cases are extremely rare. For HCV, only two cases were identified (2001 and 2005) and only one case for HIV in 2009 [17,18]. Before the mandatory implementation of HBV-NAT, 17 HBV NAT yield cases were detected. They were identified between 2007 and 2014 in the donations analysed by HBV-NAT, which covers approximately 50% of the total donations collected. From 2014 until 2021, 234 HBV positive donations were detected. Of these, 149 were HBsAg and HBV DNA-positive, whereas 76 were HBV NAT-only cases and 9 were HBsAg-only cases. Most HBV NAT yield cases were also anti-HBc positive, suggesting the presence of OBI cases. Anti-HBc donor screening is, however, not routinely performed in Switzerland.

### 3.7. Haemovigilance

For an adequate monitoring of TTI, a reliable national haemovigilance system is essential. Swissmedic publishes a haemovigilance report every 2 years. During the last 30 years, this report has described the following TTI cases: three HCV cases prior to 1992, one HIV case in 2001 and three HBV cases in 2005, 2008 and 2009.

## 4. Discussion

### 4.1. General

The infection disease safety of blood products with regard to HCV, HIV, HBV and syphilis is currently very high in Europe. Nevertheless, there are slight differences in the approaches established in various European countries. This manuscript describes the evolution of the testing strategies, the corresponding outcome of the positivity rates, the infectious disease incidences and prevalences, and the theoretical estimated RR for HCV, HIV and HBV encountered in Switzerland. It also relates to the outcome of the current testing strategy in haemovigilance reports and LB and NAT yields.

### 4.2. Blood Donations

A systematic monitoring of the three major viruses HCV, HIV, HBV and syphilis began in Switzerland in 1996. Since then, the prevalence of these infectious disease agents in FD and the incidence in RD have been collected and analysed to allow calculations on the theoretical RR. The number of blood donations collected in Switzerland has considerably declined from 572,504 in 1996 to 270,819 in 2018 but has since stabilised to approximately 270,000 per year (Figure 1). This decline is observed in other industrialised countries and is believed to be a consequence of patient blood management (PBM) protocols and demographic changes in the population. The reduction was less in FD (40,063 to 26,196), as compared to RD (532,441 to 242,006). The donations over the last 4 years have stabilised, but the percentage of FD to RD has increased from 7.5% to 10.8%, suggesting a current increase in blood product demand.

### 4.3. Evolution of the Testing Systems

All blood donations in Switzerland are currently tested for HCV, HIV and HBV with both serological methods and ID-NAT but testing for syphilis is only performed with serological methods. In contrast to the majority of other European countries, the blood donations in Switzerland are not screened for anti-HBc [19].

Serological assays for HCV, HIV and HBV and syphilis have continuously improved in both sensitivity and specificity in the last 25 years. These tests still provide the backbone of blood donation infectious disease safety. Indeed, several countries with low HCV, HIV and HBV prevalence and incidence still rely solely on serological screening (i.e., Sweden). Most other European countries, however, have introduced NAT technology to improve sensitivity and ultimately safety. The combination of serological assays and NAT is now regarded as the gold standard in most European countries [6,20,21,22]. The introduction of blood donor screening with NAT was driven in the 1990s by the AIDS and HCV epidemics, during which thousands of recipients were tragically infected with contaminated blood products and components. 

NAT screening has decreased the overall risk of TTI by reducing the WP, thus allowing the earlier detection of newly infected individuals [23,24,25]. Due to technical constraints, the first tests were conducted in up to 96 donations either manually or in semi-automated systems, targeting for HCV first and HIV-1 later. Later, sophisticated multiplexed NAT screening systems capable of detecting diverse viral variants on highly automated, high-throughput platforms were developed. These tests are highly sensitive and allowed for a dramatic reduction in the speed of analysis and sensitivity [6,7,26]. At IRB, 48 pool HCV-NAT began in 1999 with a sensitivity of 1008 IU/mL in an individual donation. Today the sensitivity is 8.3 IU/mL, which represents a 120-fold improvement. Likewise, HIV-NAT sensitivity began in 2002 with 7680 IU/mL and now resides around 25 IU/ ml, an improvement >300-fold. HBV ID-NAT was introduced 2007 in ID-NAT format with a sensitivity of 7.6 IU/mL in each individual donation and currently lies at 1.5 IU/mL (Table 1).

### 4.4. Positive Cases

As has been observed in most European countries, there has been an overall decrease over the last 25 years of confirmed positive donations in all the common blood-borne infections [9,20,21,22,27,28]. In Switzerland, the number of positive donations for the three major blood-borne viruses declined considerably between 1996 to 2021, despite reduction in the blood donations over this period. Sixty-five HCV-infected FD and fifty-four positive RD were identified in 1996. Since then, there has been a constant decline in HCV-positive FD to around eight per year in 2021, but, in contrast, there have been no single confirmed HCV positive RD since 2019. A similar HCV decline was also noticed in the general Swiss population [29]. After an initial decline, the number of HIV confirmed positive cases remained constant between 1996 and 2015, at around 4.55 confirmed HIV cases per year. Since then, however, there have only been around 1.86 cases per year identified. This decline is certainly due, in part, to the reduced number of donations collected during these two periods. Furthermore, the improved information provided to the donors has certainly reduced the number of so-called test seekers [30]. This reduction in the positive HIV donor cases is also in alignment with HIV notifications in the Swiss general population [30]. A decrease in the number of positive donors was also seen for HBV. Between 1996–1998, the number of HBV-positive RD and FD donors declined from 87 to 38. Thereafter, the decrease was only minimal, and there was even a slight peak detected in 2015/2016 due primarily to the introduction of highly sensitive HBV ID-NAT to the screening strategy. Because Swiss blood donations are not tested for anti-HBc, some additional OBI cases with extremely low viral loads, especially in RD, were detected in the years after the HBV ID-NAT introduction. However, in the general population, the number of notified HBV cases has been essentially constant over the last 25 years [31]. 

A tremendous decline in the number of detected syphilis cases shows a decrease from 56 down to 9 cases between 1996 to 2001. This reduction was especially apparent in RD. From 2002 until 2004, a notable increase was observed. This was probably due to a change of the assays used in the regional test centres. Currently, the number of positive syphilis cases fluctuates around 15 cases/year. Syphilis cases in the general population have only been noted since 2006. The FOPH has also reported a steady increase in syphilis cases since 2006 [32]. However, 88% of these syphilis cases are from men and 60% of these were reported to be from men having sex with men (MSM) and, therefore, not comparable to the blood donor situation [32].

### 4.5. NAT Yields

A number of international collaborative studies designed to capture the global usage and yield of NAT in blood donations have been completed during the last 25 years [6,33,34]. The working party of the International Society of Blood Transfusion (ISBT) on transfusion-transmitted infectious diseases (WP-TTID) performed an “international survey on NAT testing of blood donations: expanding implementation and yield from 1999–2009”. NAT data from 33 countries, covering 300 million donations, were collected and analysed. In total, 5616 HIV, HCV or HBV NAT positive donations were identified [6]. In Europe, there was a total of 829 NAT yields (73 HIV, 206 reported between 1999 and 2009 [6]). In Germany, 23 HCV, 7 HIV and 43 HBV NAT-only positives cases were identified from >30 million tested donations [35]. In 2010, Zou and colleagues reported figures for the US: 244 HCV and 32 HIV NAT-only cases within >66 million donations [36]. At that time, the US did not routinely screen with HBV-NAT.

NAT yields for Switzerland have been extremely low since the introduction of NAT screening, especially for HCV and HIV. Two HCV NAT yields per 8.2 million donations (1:4.1 million) have been identified since 1999 and only one HIV NAT yield in 2002 (1:6.8 million donations). These values appear lower than our neighbour France, who reported a NAT-yield of 1:2.0 million for HIV and 1:3.1 million for HCV between 2001–2014 [37]. In Germany between 2008–2015, similar findings have been observed (1:2.3 million for HIV and 1:756,583 for HCV) [9]. These values are slightly lower than those found in Switzerland, probably because of the difference in the NAT strategy approach of the two countries. In Germany, NAT screening is performed primarily in 96-donation pools, and thus is less sensitive than the ID-NAT approach used in Switzerland. In conclusion, these findings underscore the very low yield of HIV and HCV NAT and the advantage of the ID-NAT approach. In contrast, the number of HBV-NAT yields is higher than those for HIV and HCV. Between 2007 to 2014, 2.9 million donations were tested for HBV DNA and 17 HBV-NAT yield cases (1:170,861) were detected using different strategies and with varying sensitivities. From 2015 to 2021, 1.97 million donations were tested with ID-NAT and 74 HBV-NAT yield cases (1:25,866) were found. The majority of these donations were tested with the more sensitive cobas MPX assay on the cobas 6800/8800 platform, which has a sensitivity of 1.5 IU/mL. The distinct increase observed (from 1:170,861 to 1:25,866) can be explained by the increased detection of numerous OBI cases harbouring very low viral loads. In summary, since the introduction of HBV-NAT in 2007, when approximately 50% Swiss donations were tested, and since 2015, when all the donations were tested, a total of 4.87 million donations were screened with HBV-NAT and 91 HBV-NAT yield cases (1:53,516) were detected. This value (18.68 NAT yields/10^6^ donations) is in agreement with the figure published by Roth in 2019, who reported 16.48 HBV-NAT only cases/10^6^ donations [38]. However, in Germany, a lower HBV-NAT yield of 1:697,674 has been reported [35]. This difference is due, in part, to the different NAT testing strategy and to the fact that German blood donors are routinely screened for anti-HBc, and thus only new HBV-infected blood donors are detected.

### 4.6. Incidences/Prevalences

In parallel to the declining trend of positive donations, the incidences (Figure 3) and prevalences (Figure 4) of the three major viruses and syphilis have also declined, a situation also in accordance with the data collected from other European countries [9,20,21,22,27,28]. The reduction in HBV incidence could also be partly related to the fact that if a donor had a confirmed positive HBV marker (anti-HBc, HBV DNA, etc.), or if the donor had a positive HBV history in the past, they were permanently deferred.

### 4.7. Residual Risk (RR)

As would be expected from the reduction in HCV, HIV and HBV and syphilis contaminated blood donations, the theoretically estimated RR of transmission to blood transfusion recipients has also dramatically reduced. The development and improvement of the NAT systems over the last two decades has contributed significantly to this situation. Currently, the RR for HCV, HIV and HBV in most industrialised countries is extremely low [6,20,21,22]. The Swiss data have shown likewise that the introduction of NAT and the improvement of the technique over time has contributed to a reduction in RR, especially for HCV and HIV (Table 1). The estimated RR for Switzerland between 2019–2021 is as follows: HIV: 1:34 million donations and HCV 1:50 million donations. The figure for HBV can be divided into HBV RR including anti-HBc positive OBI cases (1:320,000) and not including the anti-HBc positive cases (1:9 million).

As shown in Table 2, the RR for HCV improved from 1:33,000 to 1:16 million donations, a gain of >480-fold (Table 2). A less but still important gain was also seen in the estimated HIV RR since the introduction of HIV-NAT and the improvement of this technology from 1:590,000 donations during 1990s to 1:11 million donations in 2021 (Table 2). In other European countries, these RR are, of course, always dependent on the specific used testing methods and incidences in a respective country. For instance, whether or not NAT is used and if so in which format, MP- or ID-NAT. In Italy, between 1996 and 2000, the RR for HIV was 1:127,000 and for HCV 1:435,000 donations, and then 15 years later from 2009–2015 it was 1:1.9 million for HIV and 1:13 million for HCV [20,21]. Spain showed a similar evolution; for HIV, 1:513,000 to 1.4 million; for HCV, 1:149,000 to 4.8 million; and for HBV, 1:74,000 to 1:346,000 from 1997–1999 and 2017, respectively [22,39]. The UK also reported similar very low RR between 2017–2019: HIV 1:25 million, HCV 1:100 million and HBV 1:1.1 million. In another study in UK, the RR for HBV was estimated to be 1:3.1 million donations with HBsAg testing and NAT testing in minipools of 24 donations [40]. In Canada, the RR for HIV was reported to be 1:36 million, which has contributed to the decision to reduce the deferral for men having sex with men to 3 months [41].

A real life confirmation of the improved safety that has occurred the last 25 years is provided LB data and the haemovigilance reports published by Swissmedic. Very few DLB and PLB have been carried out in Switzerland and those PLB that have been conducted have never confirmed a TTI (Figure 6a). The last HCV transmission occurred before 1992 and the last confirmed HIV transmission occurred in 2001, both before the introduction of NAT. Likewise, no HBV TTI has been reported since the countrywide introduction of HBV-NAT in 2010. However, previous to the implementation of HBV-NAT, two confirmed transfusion-related transmissions were reported [15,16]. The Swissmedic haemovigilance reports have described two HBV TTI cases in 2009 and 2010 and a single HIV TTI case in 2001.

Though the implementation of the NAT technology has dramatically reduced the occurrence of TTI, they have not been totally eradicated. Breakthrough cases may still occur, but, especially for HCV and HIV, these will surely be extremely infrequent. Depending on the testing strategy, the possibility of HBV TTI occurring is slightly higher but still rare. One can thus conclude a zero risk situation is an unrealistic scenario [15,16,42,43,44,45,46].

## 5. Conclusions/Outlook

The tools used to reduce TTI occurring are a combination of a selective procedures for donor recruitment, education, information and elaborated donor questionnaires. These have coincided with the introduction of various technologies used for the general screening of all blood donations; the processing of blood components, such as the leucodepletion and PRT; and post donation information (PDI). The introduction of the NAT technology for infection risks was a paramount milestone in this goal and has brought great safety to all tested blood products with regard to the major blood-borne viruses. Today we can surely say the blood products transfused in industrialised countries are extremely safe. Nevertheless, we cannot be complacent, since new emerging and re-emerging infectious disease agents are on the horizon and a continuing add-on strategy with more and more specific NAT and/or serological assays for these upcoming infectious agents is unlikely to be sustainable in the long run. There are new technologies on the horizon that may help or complement the existing technologies in the near future (i.e., digital droplet PCR, next-generation sequencing, lab-on-a-chip, and digital antigen assay). However, each of these has their limitations, either in throughput capacity, costs, automation possibilities, time to result, specificity, or the need for NAT as an integral part of the technology. Nevertheless, NAT is still the best and most efficient means for collecting results that we currently have at our disposal. With the help of risk-, effectiveness- and cost-calculation models, together with preparedness plans, it may be possible and meaningful in the near future to recalculate or redesign these approaches. With the implementation of these new measures, it should be possible to obtain a very high level of safety in blood products without increasing the respective costs. To achieve this goal, cooperation between the various stakeholders, such as blood bank establishments, manufacturers and regulators, is essential.

## Figures and Tables

**Table 1:** Evolution of the testing algorithms and platforms for the NAT testing (data for IRB as an example for Switzerland).

Virus	Year	Pool/ID	Sensitivity	Platform/Assay
HCV	Since 1999	48	1008 IU/ml	Cobas Amplicor (Roche Diagnostics) ^1^
HCV	Since 2001	48	283 IU/mL	Cobas Ampliscreen (Roche Diagnostics) ^2^
HCV	Since 2007	ID	3.7 IU/mL	Tigris/Procleix Ultrio (Chiron/Novartis)
HCV	Since 2015	ID	8.3 IU/mL	Cobas 8800/MPX (Roche Diagnostics)
HIV-1	Since 2002	48	7680 IU/mL	Cobas Ampliscreen (Roche Diagnostics) ^2^
HIV-1	Since 2007	ID	24.9 IU/mL	Tigris/Procleix Ultrio (Chiron/Novartis)
HIV-1 M HIV-1 O HIV-2	Since 2015	ID	25.7 IU/mL 8.2 IU/mL 4.0 IU/mL	Cobas 8800/MPX (Roche Diagnostics)
HBV	Since 2007	ID	7.6 IU/mL	Tigris/Procleix Ultrio (Chiron/Novartis)
HBV	Since 2015	ID	1.5 IU/mL	Cobas 8800/MPX (Roche Diagnostics)

^1^ In combination with automated extraction with QIAamp 96 Viral RNA Biorobot Kit (QIAGEN). ^2^ In combination with automated extraction with QIAamp 96 Virus Biorobot Kit (QIAGEN) on a Tecan pipetting robot.

**Table 2:** Theoretical calculated inverse of the RR for HCV, HIV and HBV from 1996 to 2021.

Year	HCV [1/RR]	HIV [1/RR]	HBV (Variant 1, Including OBI) [1/RR]	HBV (Variant 2, Excluding OBI) [1/RR]
1996	3.3 × 10^4^	5.9 × 10^5^	4.0 × 10^4^	4.0 × 10^4^
1997	2.1 × 10^5^	1.3 × 10^6^	7.9 × 10^4^	7.9 × 10^4^
1998	1.9 × 10^5^	1.5 × 10^6^	1.8 × 10^5^	1.8 × 10^5^
1999	2.3 × 10^5^	4.2 × 10^6^	9.5 × 10^4^	9.5 × 10^4^
2000	5.3 × 10^5^	4.1 × 10^6^	7.1 × 10^4^	7.1 × 10^4^
2001	3.9 × 10^6^	6.2 × 10^5^	8.4 × 10^4^	8.4 × 10^4^
2002	1.3 × 10^6^	7.3 × 10^6^	1.9 × 10^5^	1.9 × 10^5^
2003	6.5 × 10^5^	3.7 × 10^6^	1.2 × 10^5^	1.2 × 10^5^
2004	1.8 × 10^6^	1.4 × 10^6^	1.4 × 10^5^	1.4 × 10^5^
2005	3.4 × 10^6^	3.2 × 10^6^	1.3 × 10^5^	1.3 × 10^5^
2006	1.7 × 10^6^	2.1 × 10^6^	2.5 × 10^5^	2.5 × 10^5^
2007	6.9 × 10^5^	1.7 × 10^6^	1.3 × 10^5^	1.3 × 10^5^
2008	∞	9.3 × 10^6^	1.3 × 10^5^	1.3 × 10^5^
2009	1.1 × 10^7^	4.6 × 10^6^	5.2 × 10^5^	5.2 × 10^5^
2010	1.4 × 10^7^	2.4 × 10^6^	4.3 × 10^5^	4.3 × 10^5^
2011	6.4 × 10^6^	4.6 × 10^6^	3.4 × 10^5^	3.4 × 10^5^
2012	4.5 × 10^6^	9.7 × 10^6^	4.3 × 10^5^	4.3 × 10^5^
2013	1.2 × 10^7^	8.3 × 10^6^	∞	∞
2014	1.4 × 10^7^	3.3 × 10^6^	7.3 × 10^5^	7.3 × 10^5^
2015	1.5 × 10^7^	∞	3.4 × 10^5^	3.4 × 10^5^
2016	9.4 × 10^6^	6.3 × 10^6^	1.7 × 10^5^	8.4 × 10^5^
2017	1.9 × 10^7^	6.3 × 10^6^	3.4 × 10^5^	∞
2018	1.6 × 10^7^	1.1 × 10^7^	3.2 × 10^5^	2.8 × 10^6^
2019	∞	∞	3.9 × 10^5^	∞
2020	∞	∞	2.3 × 10^5^	3.0 × 10^6^
2021	∞	1.1 × 10^7^	4.2 × 10^5^	∞

HCV until June 1999 without NAT, HIV until February 2002 without NAT, HBV from 2007 partly with NAT. The theoretical risk estimations were carried out for HBV with the NAT windows since the introduction of NAT in 2011. For HBV, since 2016 both scenarios (with and without the OBI cases) are modelled. ∞ no positive case in the corresponding year.

## Data Availability

Not applicable.

## Appendix A

**Table A1:** Theoretical calculated inverse of the RR for HCV, HIV and HBV from 1996 to 2021, including the 95% confidence intervals.

	HCV			HIV			HBV (Variant 1, Including OBI)			HBV (Variant 2, Excluding OBI)		
Year	1/RR	95% Confidence Interval		1/RR	95% Confidence Interval		1/RR	95% Confidence Interval		1/RR	95% Confidence Interval	
1996	3.3 × 10^4^	2.5 × 10^4^	4.4 × 10^4^	5.9 × 10^5^	3.1 × 10^5^	1.3 × 10^6^	4.0 × 10^4^	2.6 × 10^4^	6.4 × 10^4^	4.0 × 10^4^	2.6 × 10^4^	6.4 × 10^4^
1997	2.1 × 10^5^	1.1 × 10^5^	4.9 × 10^5^	1.3 × 10^6^	4.9 × 10^5^	4.6 × 10^6^	7.9 × 10^4^	4.3 × 10^4^	1.7 × 10^5^	7.9 × 10^4^	4.3 × 10^4^	1.7 × 10^5^
1998	1.9 × 10^5^	9.7 × 10^4^	4.4 × 10^5^	1.5 × 10^6^	5.3 × 10^5^	7.4 × 10^6^	1.8 × 10^5^	7.0 × 10^4^	6.6 × 10^5^	1.8 × 10^5^	7.0 × 10^4^	6.6 × 10^5^
1999	2.3 × 10^5^	1.5 × 10^5^	3.9 × 10^5^	4.2 × 10^6^	7.6 × 10^5^	1.7 × 10^8^	9.5 × 10^4^	4.6 × 10^4^	2.4 × 10^5^	9.5 × 10^4^	4.6 × 10^4^	2.4 × 10^5^
2000	5.3 × 10^5^	2.7 × 10^5^	1.2 × 10^6^	4.1 × 10^6^	7.3 × 10^5^	1.6 × 10^8^	7.1 × 10^4^	3.7 × 10^4^	1.6 × 10^5^	7.1 × 10^4^	3.7 × 10^4^	1.6 × 10^5^
2001	3.9 × 106	7.0 × 10^5^	1.5 × 10^8^	6.2 × 10^5^	2.9 × 10^5^	1.7 × 10^6^	8.4 × 10^4^	4.1 × 10^4^	2.1 × 10^5^	8.4 × 10^4^	4.1 × 10^4^	2.1 × 10^5^
2002	1.3 × 10^6^	4.4 × 10^5^	6.2 × 10^6^	7.3 × 10^6^	1.3 × 10^6^	2.9 × 10^8^	1.9 × 10^5^	6.5 × 10^4^	9.3 × 10^5^	1.9 × 10^5^	6.5 × 10^4^	9.3 × 10^5^
2003	6.5 × 10^5^	3.0 × 10^5^	1.8 × 10^6^	3.7 × 10^6^	1.0 × 10^6^	3.1 × 10^7^	1.2 × 10^5^	5.0 × 10^4^	3.6 × 10^5^	1.2 × 10^5^	5.0 × 10^4^	3.6 × 10^5^
2004	1.8 × 10^6^	5.1 × 10^5^	1.5 × 10^7^	1.4 × 10^6^	6.0 × 10^5^	4.3 × 10^6^	1.4 × 10^5^	5.4 × 10^4^	5.1 × 10^5^	1.4 × 10^5^	5.4 × 10^4^	5.1 × 10^5^
2005	3.4 × 10^6^	6.1 × 10^5^	1.3 × 10^8^	3.2 × 10^6^	9.0 × 10^5^	2.7 × 10^7^	1.3 × 10^5^	5.0 × 10^4^	4.7 × 10^5^	1.3 × 10^5^	5.0 × 10^4^	4.7 × 10^5^
2006	1.7 × 10^6^	4.6 × 10^5^	1.4 × 10^7^	2.1 × 10^6^	7.3 × 10^5^	1.0 × 10^7^	2.5 × 10^5^	6.9 × 10^4^	2.1 × 10^6^	2.5 × 10^5^	6.9 × 10^4^	2.1 × 10^6^
2007	6.9 × 10^5^	3.0 × 10^5^	2.1 × 10^6^	1.7 × 10^6^	6.5 × 10^5^	6.1 × 10^6^	1.3 × 10^5^	5.1 × 10^4^	4.8 × 10^5^	1.3 × 10^5^	5.1 × 10^4^	4.8 × 10^5^
2008	∞	2.9 × 10^6^	∞	9.3 × 10^6^	1.7 × 10^6^	3.7 × 10^8^	1.3 × 10^5^	6.6 × 10^4^	3.0 × 10^5^	1.3 × 10^5^	6.6 × 10^4^	3.0 × 10^5^
2009	1.1 × 10^7^	1.9 × 10^6^	4.2 × 10^8^	4.6 × 10^6^	1.3 × 10^6^	3.8 × 10^7^	5.2 × 10^5^	1.4 × 10^5^	4.3 × 10^6^	5.2 × 10^5^	1.4 × 10^5^	4.3 × 10^6^
2010	1.4 × 10^7^	2.4 × 10^6^	5.4 × 10^8^	2.4 × 10^6^	9.5 × 10^5^	8.9 × 10^6^	4.3 × 10^5^	1.8 × 10^5^	1.3 × 10^6^	4.3 × 10^5^	1.8 × 10^5^	1.3 × 10^6^
2011	6.4 × 10^6^	1.8 × 10^6^	5.3 × 10^7^	4.6 × 10^6^	1.3 × 10^6^	3.8 × 10^7^	3.4 × 10^5^	1.6 × 10^5^	9.2 × 10^5^	3.4 × 10^5^	1.6 × 10^5^	9.2 × 10^5^
2012	4.5 × 10^6^	1.5 × 10^6^	2.2 × 10^7^	9.7 × 10^6^	1.7 × 10^6^	3.8 × 10^8^	4.3 × 10^5^	1.8 × 10^5^	1.3 × 10^6^	4.3 × 10^5^	1.8 × 10^5^	1.3 × 10^6^
2013	1.2 × 10^7^	2.1 × 10^6^	4.6 × 10^8^	8.3 × 10^6^	1.5 × 10^6^	3.3 × 10^8^	∞	5.0 × 10^5^	∞	∞	5.0 × 10^5^	∞
2014	1.4 × 10^7^	2.5 × 10^6^	5.4 × 10^8^	3.3 × 10^6^	1.1 × 10^6^	1.6 × 10^7^	7.3 × 10^5^	2.5 × 10^5^	3.5 × 10^6^	7.3 × 10^5^	2.5 × 10^5^	3.5 × 10^6^
2015	1.5 × 10^7^	2.7 × 10^6^	5.8 × 10^8^	∞	2.9 × 10^6^	∞	3.4 × 10^5^	1.6 × 10^5^	8.3 × 10^5^	3.4 × 10^5^	1.6 × 10^5^	8.3 × 10^5^
2016	9.4 × 10^6^	2.6 × 10^6^	7.8 × 10^7^	6.3 × 10^6^	1.7 × 10^6^	5.2 × 10^7^	1.7 × 10^5^	1.1 × 10^5^	2.7 × 10^5^	8.4 × 10^5^	3.3 × 10^5^	3.1 × 10^6^
2017	1.9 × 10^7^	3.4 × 10^6^	7.5 × 10^8^	6.3 × 10^6^	1.7 × 10^6^	5.2 × 10^7^	3.4 × 10^5^	1.8 × 10^5^	7.0 × 10^5^	∞	9.1 × 10^5^	∞
2018	1.6 × 10^7^	2.9 × 10^6^	6.3 × 10^8^	1.1 × 10^7^	1.9 × 10^6^	4.2 × 10^8^	3.2 × 10^5^	1.7 × 10^5^	6.9 × 10^5^	2.8 × 10^6^	5.1 × 10^5^	1.1 × 10^8^
2019	∞	4.7 × 10^6^	∞	∞	3.1 × 10^6^	∞	3.9 × 10^5^	2.0 × 10^5^	9.0 × 10^5^	∞	8.4 × 10^5^	∞
2020	∞	4.6 × 10^6^	∞	∞	3.1 × 10^6^	∞	2.3 × 10^5^	1.4 × 10^5^	4.3 × 10^5^	3.0 × 10^6^	5.4 × 10^5^	1.2 × 10^8^
2021	∞	4.5 × 10^6^	∞	1.1 × 10^7^	2.0 × 10^6^	4.3 × 10^8^	4.2 × 10^5^	2.0 × 10^5^	1.0 × 10^6^	∞	7.9 × 10^5^	∞
